# A Solar-Driven Flexible Electrochromic Supercapacitor

**DOI:** 10.3390/ma13051206

**Published:** 2020-03-09

**Authors:** Danni Zhang, Baolin Sun, Hui Huang, Yongping Gan, Yang Xia, Chu Liang, Wenkui Zhang, Jun Zhang

**Affiliations:** College of Materials Science and Engineering, Zhejiang University of Technology, Hangzhou 310014, Chinahhui@zjut.edu.cn (H.H.); ganyp@zjut.edu.cn (Y.G.); nanoshine@zjut.edu.cn (Y.X.); cliang@zjut.edu.cn (C.L.); msechem@zjut.edu.cn (W.Z.)

**Keywords:** photoelectrochromic devices (PECD), dye-sensitized solar cells (DSSC), electrochromic devices (ECD), smart windows, flexible devices

## Abstract

Solar-driven electrochromic smart windows with energy-storage ability are promising for energy-saving buildings. In this work, a flexible photoelectrochromic device (PECD) was designed for this purpose. The PECD is composed of two flexible transparent conductive layers, a photocatalytic layer, an electrochromic material layer, and a transparent electrolyte layer. The photocatalytic layer is a dye-sensitized TiO_2_ thick film and the electrochromic layer is a WO_3_ thin film, which also possesses a supercapacitive property. Under illumination, dye-sensitized TiO_2_ thick film realizes photo-drive electrochromism that the WO_3_ changes from colorless to blue with large optical modulation. Meanwhile, the PECD has an electrochemical supercapacitance showing an energy storage property of 21 mF·cm^−2^ (114.9 F·g^−1^ vs the mass of WO_3_), stable mechanical performance and long cycle performance. The PECD can effectively adjust the transmittance of visible and near-infrared light without any external power supply, realizing zero energy consumption, and can convert solar energy into electrical energy for storage.

## 1. Introduction

The negative impact of traditional energy consumption on the environment has aroused great social concerns. According to relevant calculations, the annual energy consumption of buildings accounts for about 40% of the world’s total energy consumption [1]. In addition, doors and windows are the most serious parts of a building’s energy loss and their energy consumption accounts for a large proportion of the total energy consumption of buildings [2]. Currently, the widely used low-emission (Low-E) glass can limit the heat exchange between the indoor and the outdoor environments [3], but it cannot realize the continuous regulation of light. Therefore, it is urgent to develop new green smart windows with low energy consumption and a large optical modulation range.

Electrochromic devices (ECDs) can produce a reversible color change when charge insertion/extraction or chemical reduction/oxidation processes occur under electrochemical stimulation [4,5,6]. The features of ECDs make it have great application potential not only in smart windows [7,8,9], but also in anti-glare rearview mirrors [10], displays [11] and encryption devices [12]. In order to obtain a better performance, including contrast ratio [13], coloration efficiency (CE) [14], response time [15] and cycle life [16], a transparent electrode is required to have a low sheet resistance, high transmittance, stable electrochemical performance, stable mechanical performance and a high Figure of Merit (FoM, the ratio of electrical conductivity $\sigma_{CV}$ and optical conductivity $\sigma_{OP}$, $\sigma_{CV}$/$\sigma_{OP}$) [17]. Traditional electrodes are rigid, such as indium tin oxide (ITO) [18] or fluorine-doped tin oxide (FTO) [19] glass. However, their applications are limited by their disadvantages, such as their high price, their being difficult to carry and their unbending properties. Therefore, researchers pay attention to flexible ECDs [20,21] made with flexible transparent electrodes. For example, ITO [22], metallic nanowires/grids (Ag, Au) [23,24], carbon nanotubes [25], graphene [26], and conductive polymers [27] on flexible substrates. ITO, with its high transmittance and low sheet resistivity, has become one of the most common electrodes. In addition, electrochromic materials are very significant for ECDs. Transition metal oxide WO_3_ [28,29,30] has been widely studied due to its excellent electrochemical and electrochromic properties. With the intercalating of metal cation, W^6+^ is reduced to W^5+^ and W^4+^, causing the color to change from colorless to blue.

However, conventional ECDs do not change color automatically and require an external power source to achieve electrochromism [31,32], which is not easy to carry and is associated with energy consumption. Solar cells can convert solar energy into electric energy and are environmentally friendly. By connecting solar cells with ECDs, photoelectrochromism can be realized [33]. The basic components of photo-drive ECDs mainly include photovoltaic modules and electrochromic modules, which are roughly divided into two types. In the first type, photovoltaic modules and electrochromic energy storage modules are independent of each other and are connected by external circuits. Photovoltaic modules convert solar energy into electric energy so that they can charge for electrochromic modules and drive the color changing, which can be referred to as photovoltaic electrochromic devices (PV-ECDs). For example, Xia et al. [34] connected perovskite solar cells with electrochromic devices with wires so that solar cells could supply power to ECDs, realizing solar energy capture, electrochemical energy storage, electrochromism and recycling. In the second type, photovoltaic modules and electrochromic energy storage modules are integrated into one device, and the electrolyte is shared by the two modules. No external circuit connection is required, simplifying the device structure, which can be referred to as photoelectrochromic device (PECDs). For example, Leftheriotis et al. [35,36] designed “Partly covered” photoelectrochromic devices by integrating dye-sensitized solar cells and an ECD into one device, which showed enhanced coloration speed and efficiency. Xu et al. [37] designed an optically driven ECD, which integrates a large area of electrochromic parts with several small fiber-like DSSCs, and can optimize the optical drive power by means of series or parallel photovoltaic modules to achieve photo-driven electrochromism. In addition, Tong et al. studied [38] the possibility of integration between electrochromic devices and photovoltaic devices and structures for photoelectrochromic devices. However, photoelectrochromic devices studied are almost always rigidly based on FTO/ITO glass and flexible devices are rare.

In this work, we combined the DSSC with the ECD based on WO_3_ electrochromic material in a horizontal way to form a flexible PECD. Our structure is not only simpler and more integrated than the traditional ECDs, but also does not require an external power source. The DSSC module will convert the solar energy into electricity to charge the ECD module under illumination and achieve photoelectrochromism realizing zero energy consumption. It provides solutions for achieving low-energy green buildings. Compared with ordinary windows, this PECD can intelligently adjust the transmittance of the visible band and near-infrared band in the sunlight, thus effectively regulating indoor visibility and temperature. Moreover, this flexible feature can make it suitable for various shapes of glass and any place. In addition, the electrochromic module can also store energy and be used as a supercapacitor.

## 2. Materials and Methods

### 2.1. Materials and Reagents

Indium tin oxide-polyethylene glycol terephthalate (ITO-PET) substrates (sheet resistance 35 Ω·sq^−1^, transmittance > 82%) were purchased from Zhuhai Kaivo Optoelectronic Technology Co., Ltd. (Zhuhai, China). W target was purchased from Hefei Kejing Materials Technology Co., Ltd. (Hefei, China). Iodine (AR, 99.8%), lithium iodide (99%) and propylene carbonate (PC, 99.7%) were purchased from Aladdin (Shanghai, China). Di-tetrabutylammonium cis-bis (isothiocyanato) bis (2,2′-bipyridyl-4,4′-dicarboxylato) ruthenium (II) (N719, > 95%) and surlyn sealing films (60 μm) were purchased from Opv-Tech Co., Ltd. (Yingkou, China). N,N-dimethylformamide (DMF, 99.5%) was purchased from Xilong Scientific Co., Ltd. (Shantou, China). Tetrabutyl titanate (CP, 98.0%) was purchased from Shanghai Lingfeng Chemical Reagent Co., Ltd. (Shanghai, China). Poly (vinylidene fluoride-co-hexafluoro propylene) (PVDF-HFP, Arkema 2801) and TiO_2_ (Degussa P25) were used without further treatment.

### 2.2. Fabrication of the Photoanode and Counter Electrode

The commercial ITO-PET substrates were first cleaned with ethyl alcohol and deionized water sequentially. The precleaned substrates were then dried in the oven. The TiO_2_ film was prepared by the doctor-blade method; the specific steps are as follows: First, 1 g TiO_2_ powder and several drops of tetrabutyl titanate were added into 4.4 mL ethyl alcohol with stirring for 24 h. Second, tape was pasted on both sides of the reserved DSSC part and excessive TiO_2_ colloid was dropped, and then manually scraped with the smooth side of FTO glass until the surface of the TiO_2_ film was smooth and uniform. After the ethanol was volatilized at room temperature, the by-product alcohols were removed by heat treatment at 120 °C for 2 h. Finally, 20 MPa pressure was applied to increase the adhesion between the TiO_2_ film and the substrate.

Pt electrode was obtained by vacuum sputtering for 45s, presenting a transparent light gray color.

### 2.3. Preparation of WO_3_ Thin Film

The WO_3_ film was coated on the same ITO-PET substrate by the DC magnetron sputtering method, parallel to TiO_2_ film. First, the TiO_2_ film prepared above was covered by a mask to prevent coating the WO_3_. Then, the WO_3_ film was prepared in an atmosphere of pure argon and oxygen gases with a flow ratio of 2:1, power of 115 W, pressure of 0.7 Pa and sputtering time of 40 min. Finally, the TiO_2_/WO_3_/ITO-PET electrode was immersed into 0.5 mM N719 in ethanol for 24 h.

### 2.4. Fabrication of the Electrolytes

Preparation of the membrane: Firstly, PVDF-HFP was dissolved in DMF solvent with a mass ratio of 3:7 and heated at 60 °C for 24 h to fully dissolve PVDF-HFP. Then, PVDF-HFP was scraped evenly on the glass and transferred into water to obtain a film. Finally, it was put into a vacuum oven at 80 °C to dry.

Preparation of Electrolyte: 0.5 M LiI and 0.005 M I_2_ were dissolved in PC solvent and stirred for 24 h. The prepared membrane was cut to a suitable size and soaked in electrolyte for 24 h.

### 2.5. Fabrication of the PECD

PECDs are composed of the WO_3_/TiO_2_/ITO-PET electrode, electrolyte and a Pt counter electrode. First, a suitably sized electrolyte membrane was applied to the WO_3_/TiO_2_/ITO-PET electrode, followed by a surlyn sealing film around the electrolyte membrane, and then a Pt electrode was covered. Second, copper tape was attached to the side of the ITO-PET and pulled out for electrical conductivity. At last, it was put into card films and overplasticized.

### 2.6. Characterization

The structural properties of WO_3_ films were characterized by X-ray diffraction (XRD, Rigaku Ultima IV, Tokyo, Japan). The surface morphology and crossing morphology of the films were characterized by field emission scanning electron microscopy (FESEM, Hitachi S4700, Tokyo, Japan). The ultraviolet-visible-near-infrared (UV-VIS-NIR) transmission spectra of the assembled devices were characterized using a UV-3600 spectrophotometer. The electrochemical performance of the assembled devices was characterized by the Zennium electrochemical workstation (ZAHNER, Kronach, Germany).

## 3. Results and Discussion

**Figure 1:**
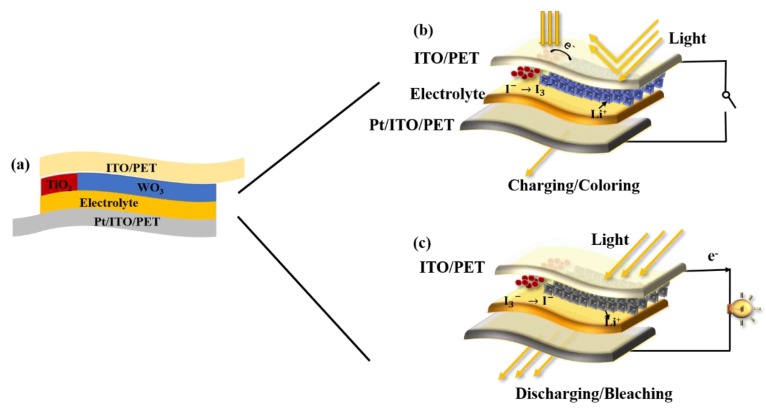
Schematic diagrams of the photoelectrochromic device (PECD); (**a**) photo-charging process of the PECD and (**b**) the discharging process of the PECD (**c**).

The structural diagram and schematic diagram are shown in Figure 1. The PECD has a sandwich structure [39] consisting of the DSSC module and the ECD module. In Figure 1a, the DSSC module is composed of the ITO-PET electrode, electrolyte, TiO_2_ film and the Pt electrode, and the ECD module is composed of the ITO-PET electrode, electrolyte, WO_3_ film and the Pt electrode, which share the ITO-PET electrode, electrolyte and the Pt electrode. The size of the electrode is 3.5 cm × 2 cm, in which the magnetron sputtering WO_3_ film is 2 cm × 2 cm, the TiO_2_ film is 0.5 cm × 2cm, and the extra part was used for sticking copper tape. Figure 1b,c illustrates the coloring and bleaching principle of the PECD, respectively. When the device is exposed to the light, the dye molecules are excited to generate electron hole pairs, and the electrons are injected into the TiO_2_ conduction band, and then diffused to the ITO-PET substrate and WO_3_. In order to neutralize the electrons enriched in WO_3_, Li^+^ in electrolyte is intercalated in WO_3_, and redox reaction occurs, causing WO_3_ to turn from colorless to blue. The PECD in this state can block most incident light. At the same time, the dye loses electrons and is reduced by I^−^ to realize dye regeneration. When the device is in short circuit or connected with external electrical appliances, the co-deintercalation of electrons and Li^+^ occurs, making WO_3_ change from blue to colorless. The PECD in this state allows most of the incident light to pass through. At the same time, the oxidized electrolyte is reduced after receiving electrons at the Pt electrode, thus completing the cycle. The schematic reaction can be summarized as the equation below:

(1) $${\left[{\mathrm{WO}}_{3}+{\mathrm{xLi}}^{+}+{\mathrm{xe}}^{-}\right]}_{\mathrm{bleached}}↔{\left[{\mathrm{Li}}_{x}{\mathrm{WO}}_{3}\right]}_{\mathrm{colored}}$$

(2) $$I^{-}↔I_{3}{}^{-}$$

**Figure 2:**
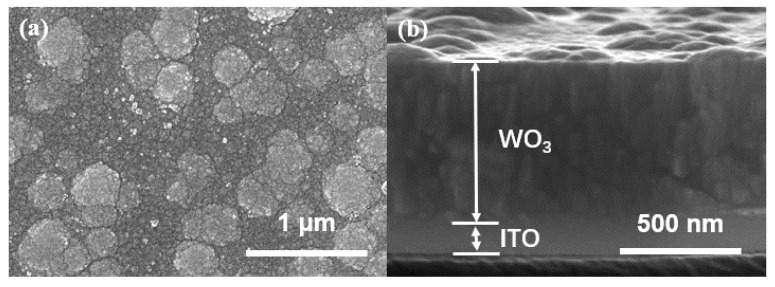
(**a**) Field emission scanning electron microscopy (FESEM) images of the WO_3_; (**b**) FESEM image of the thickness of the WO_3_.

**Figure 3:**
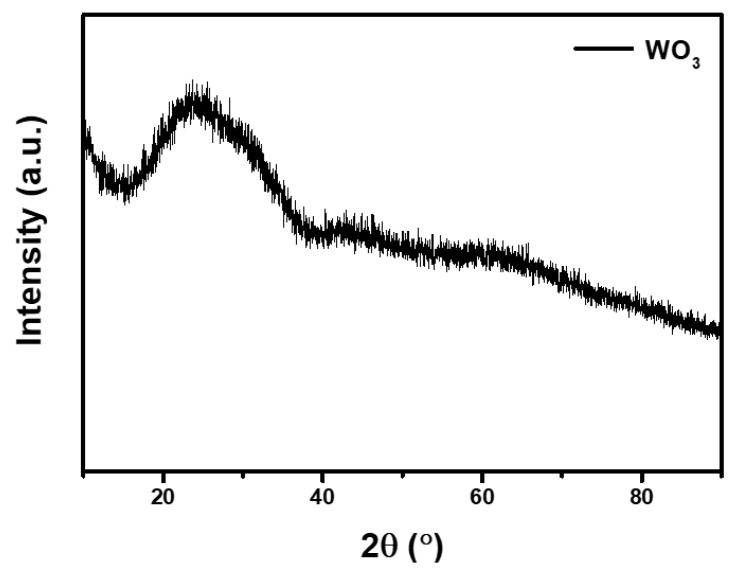
XRD pattern of the obtained WO_3_.

To better understand the structures and morphology of WO_3_ films, FESEM and XRD tests were performed. The surface morphology of WO_3_ films is shown in Figure 2a. WO_3_ is distributed compactly on the substrate and agglomerated. The thickness of the sputtered WO_3_ over ITO-PET is estimated at about 600 nm, as shown in Figure 2b. In Figure 3, XRD has no characteristic peak of WO_3_, indicating that WO_3_ obtained by magnetron sputtering is amorphous. Amorphous tungsten oxide films usually have a higher coloration efficiency and faster switching time [40,41].

**Figure 4:**
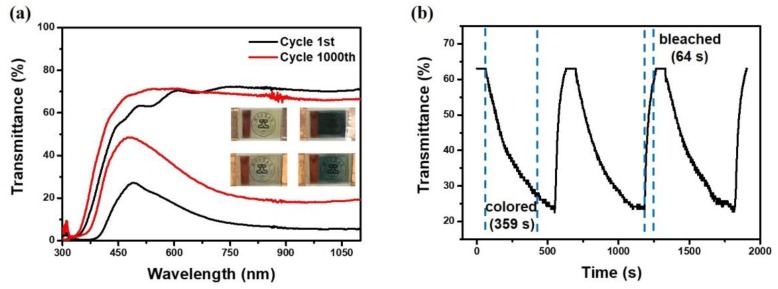
(**a**) The transmittance spectrum of the PECD in 300–1100 nm at the 1st cycle and the 1000th cycle; the insert corresponds to the 1st cycle bleaching and coloring state and the 1000th cycle bleaching and coloring state; (**b**) switching performance of the PECD at 529 nm.

In the PECD, the DSSC shares the same electrolyte with the ECD, so it is important to design a suitable electrolyte to support them. To get a higher ionic conductivity of the electrolyte, we prepared a PVDF-HFP membrane infiltrating liquid electrolyte and gel electrolyte with PVDF-HFP as gels. Supplementary Materials Figure S1 shows EIS of the PVDF-HFP membrane infiltrating liquid electrolyte and PVDF-HFP gel electrolyte, and the former is about 270 Ω, smaller than the gel electrolyte, so the PECD with this PVDF-HFP membrane shows a better electrochemical and electrochromic performance. However, compared with the traditional DSSC, the ionic conductivity is still not ideal because the electrolyte concentration is reduced in order to increase the transmittance. As shown in Figure 4a, the transmittance spectra of the bleached state and the colored state of the PECD were tested within the wavelength range of 300–1100 nm. The transmittance of the colored state was tested after being exposed to a 1000 W xenon lamp for 10 min and the transmittance of the bleached state was tested after applying −1 V voltage for 3 min. Compared with the bleached state, the transmittance of the PECD in the colored state decreases significantly, which can effectively block out near-infrared and visible light to regulate temperature and indoor visibility, indicating that it has great application potential in the field of smart windows. After 1000 cycles, the transmittance of the bleached state changes slightly, while that of the colored state increases significantly. The colored and bleached images of the first cycle and the 1000th cycle are shown in the illustration in Figure 4a. The precise transmittance values at 529 nm and 860 nm of the PECD are given in Table S1. Since the electrolyte is yellowish in color, the overall appearance of the device in the bleached state is yellowish. From Figure S2, we can see the photo of the PVDF-HFP membrane before and after the electrolyte immersion. The newly prepared membrane is thin and smooth, showing a white color. After electrolyte immersion, the membrane changes from white to transparent yellow and the transmittance increases significantly, so the optical effect on the electrolyte is negligible.

Switching time is usually an important parameter for ECDs and is defined as the spanning time required for a 90% change between the bleached and colored states. Green light at 529 nm was used as the light source, and the photosensitive resistor was used as the original test element. The light source, PECD, and photosensitive resistor were arranged in order at the same horizontal line. The DSSC module of the PECD was exposed while the ECD module, the light source and the photosensitive resistor were in the dark state. The relationship between the current and the time recorded by the photosensitive resistor was converted into the relationship between the transmittance (at 529 nm) and time so as to obtain the switching time. The switching time of the PECD is shown in Figure 4b; the coloring time is slower than that of traditional electrochromic devices, 359 s, and the bleaching time is 64 s when −1 V voltage is applied. Four guesses about the slower switching time than traditional ECD are as follows: The impedance of the electrolyte is shown in the Figure S1, and the ionic conductivity of the electrolyte is not high. The J–V curve of the flexible DSSC is shown in Figure S3a, the short-circuit current density is 0.21 mA/cm^2^, the open-circuit voltage is 0.63 V, and the conversion efficiency is not ideal. The J–V curve of the PECD is shown in Figure S3b, the open circuit voltage is about 0.6 V and although it can drive WO_3_ from colorless to blue, it is low compared with the voltage applied by electrochemical workstations in other studies [42,43]. And it takes time for the PECD to reach sufficient voltage. In addition, compared with the traditional DSSC, the short-circuit current density of the PECD is not the maximum, which may be due to the addition of the ECD module, which undergoes redox reaction.

**Figure 5:**
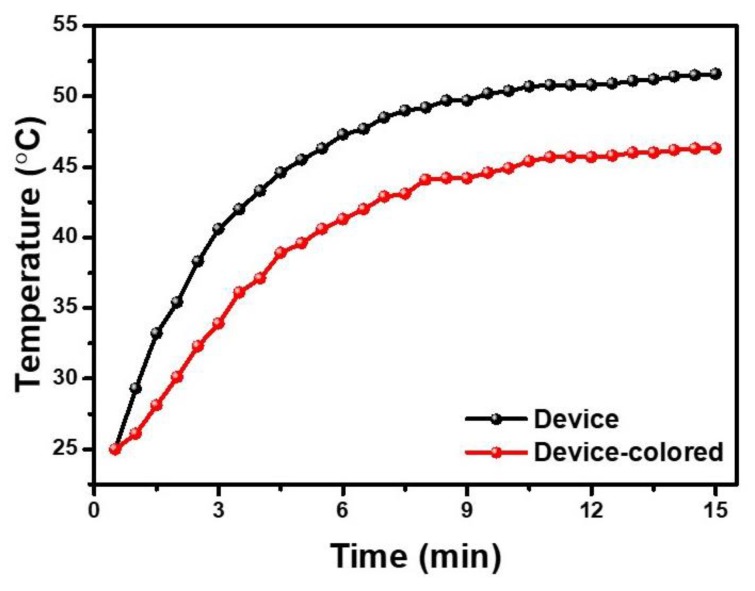
Temperature of the two rooms versus the infrared irradiation time, the windows of the room are made up of different devices.

Since the PECD of this structure can realize the optical regulation of near-infrared light, a model house was prepared to study the temperature control effect of the PECD. To prove that this PECD has the effect of temperature control compared with ordinary devices, the PECD in colored state and the WO_3−_ free device act as the window of the model house. As shown in Figure 5, the indoor temperature of the model house increases significantly when exposed to the infrared lamp. After 10 min, the temperature rises at a steady rate. The indoor temperature of the window with the colored PECD is always lower than that of the window without WO_3_. In 15 min, the indoor temperature increases from room temperature to 46.3 °C, while the other one increases from room temperature to 51.6 °C, which strongly proves that the PECD as a smart window has a good temperature control effect. Therefore, when the PECD is applied in real life, it can automatically change from a bleached state to a colored state on sunny days, reducing the transmittance of near-infrared light and achieving the effect of temperature control. In addition, the PECD can be changed from a colored to a bleached state by applying voltage when light is needed in the room.

**Figure 6:**
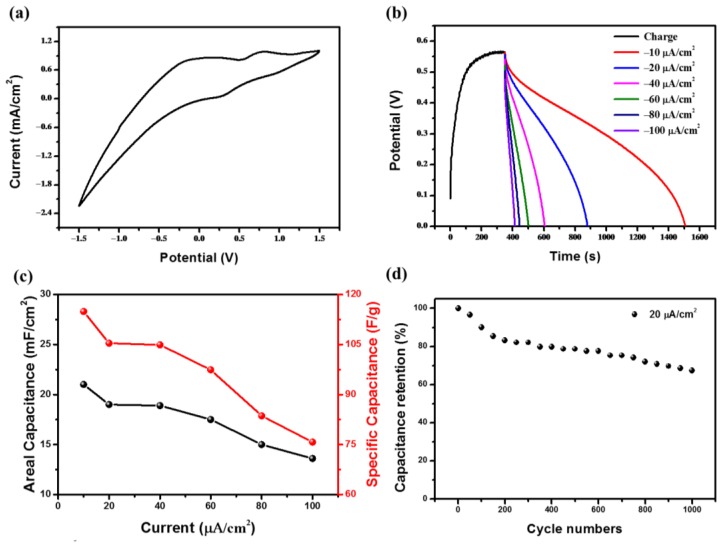
(**a**) The cyclic voltammetry curve of the PECD; (**b**) photo-charge curve and galvanostatic discharge curves of the PECD; (**c**) areal capacitances and specific capacitances of the WO_3_ film at different current densities; (**d**) cycle performance of the PECD under a current density of 20 μA/cm^2^.

The PECD can not only realize photo-drive electrochromic performance, showing excellent electrochromic performance, but also has a good electrochemical performance. Figure 6a shows the C–V curve of the PECD between −1.5 V and 1.5 V at the scan rate of 100 mV s^−1^ in the dark. Two redox pairs are found in the CV curve, corresponding to Li_x_WO_3_/WO_3_ and I_3_^−^/I^−^, respectively. Figure 6b shows the photo charge curve of the PECD and the constant current discharge curves at the current density of 10, 20, 40, 60, 80, 100 μA·cm^−2^. The device can reach an open circuit voltage of 0.55 V at a light intensity of 1000 W·m^−2^ and the discharge time decreases with the increase of discharge current density. As an energy storage device, capacitance is very important to it. Areal capacitance and the specific capacitance of the PECD are shown in Figure 6c, and both areal capacitance and specific capacitance decrease with the increase of discharge current density. At 10 μA·cm^−2^, the maximum capacitance is 21 mF·cm^−2^ (114.9 F·g^−1^) and the minimum capacitance is 13.6 mF·cm^−2^ (75.7 F·g^−1^) at 100 μA·cm^−2^. Another important performance of the PECD is cycle stability. Figure 6d shows the capacitance retention of the PECD after 1000 cycles at 20 μA·cm^−2^ discharge current density. In the first two hundred cycles of the PECD, the capacitance decays rapidly, and in the later stage, it is relatively stable and drops slowly. Our guess is that there was some residual Li^+^ in WO_3_ during the initial Li^+^ insertion and extraction process, which led to this result. In addition, a previous study has shown that the cycling ability of crystalline WO_3_ is better than that of amorphous WO_3_ [44]. After 1000 cycles, the capacitance is about 65% of the initial value. In addition, the PECD can be used as an energy storage device. As a conceptual demonstration, several PECDs are connected in a series to act as a power supply in Figure S4. When the PECDs are fully charged, the devices are deep blue and light up red LEDs.

**Figure 7:**
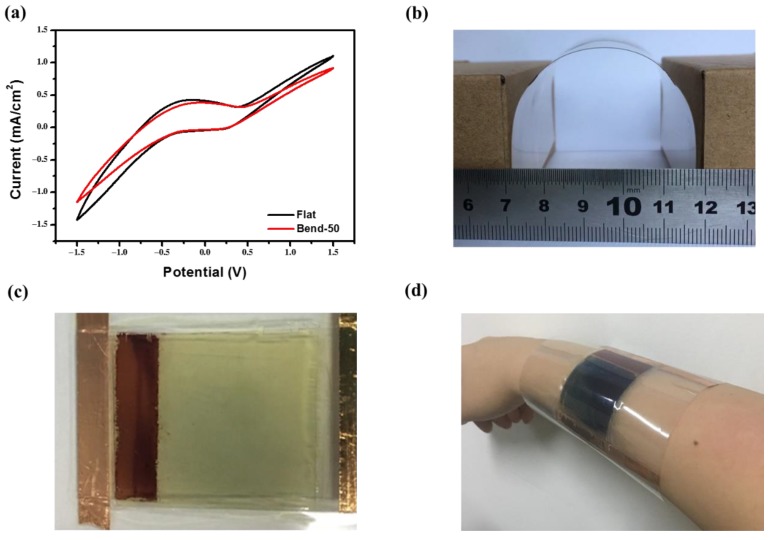
(**a**) The cyclic voltammetry curve of the PECD at the flat state and after 50 bends; (**b**) photograph of the bend state; (**c**) photograph of the enlarged PECD at the bleached state; (**d**) photograph of the enlarged PECD in the wearable field.

The substrate of this device is ITO-PET. In contrast to traditional ITO and FTO glass, ITO-PET not only has a low sheet resistance and high transmittance, but also has excellent flexibility. In order to study the bending stability of the PECD, the PECD was bent, and the bending state is shown in Figure 7b. The C–V curve of the PECD at the initial state and after bending 50 times was tested. As shown in Figure 7a, there is a slight difference between the CV curves, indicating that the PECD has a great bending stability. The amplification of the PECD has an enlightening effect on the later practical study. Now, the PECD is scaled up to four times. Figure 7c shows the bleached state of the enlarged PECD composed of the DSSC module on the left and the ECD module on the right. In addition, Figure 7d shows the PECD on the human hand. In the bending state, the enlarged PECD can still be automatically colored under illumination, indicating its bending stability. Due to the smoothness and flexibility of the device, it can also be used in wearable applications. Furthermore, it is possible to implement smart windows of any shape and keep good performance.

## 4. Conclusions

In summary, we successfully integrated the DSSC with the ECD using WO_3_ as electrochromic material to prepare a flexible PECD which shows excellent electrochromic performance and electrochemical performance. The switching time of the PECD is 359 s (coloring under illumination) and 64 s (bleaching under −1 V voltage), respectively. Moreover, the optical modulation range of near-infrared light reaches more than 60%. The maximum capacitance can reach 21 mF·cm^−2^ (114.9 F·g^−1^) and several PECDs connected in series can light up LEDs. At the same time, the enlarged device still exhibits a similar performance. However, due to the lack of understanding of the energy level matching of each functional component and photochemical reaction mechanism, PECDs are still rare, so the model needs to be further studied in order to realize the practical applications of the device in smart windows and other fields.

## Supplementary Materials

The following are available online at https://www.mdpi.com/1996-1944/13/5/1206/s1. Figure S1. EIS spectra of the liquid electrolyte and gel electrolyte; Figure S2. The photograph of PVDF-HFP membrane before and after soaking electrolyte; Figure S3. J-V curves of DSSC (a) and PECD (b) under the irradiation of 1000 W/m^2^; Figure S4. “ZJUT” composed by several red LEDs lit up by connected PECDs; Table S1. Transmittance properties of PECD at 529 nm and 860 nm.
