# Supplementary material for: A Solar-Driven Flexible Electrochromic Supercapacitor
Source: Materials (Basel). 2020 Mar 9;13(5):1206. doi: 10.3390/ma13051206 (PMC7085080; doi:10.3390/ma13051206)
Supplement: Supplementary file 1 [file materials-13-01206-s001.pdf]

materials

MDPI

Supplementary Materials

A Solar-Driven Flexible Electrochromic Supercapacitor

Danni Zhang, Baolin Sun, Hui Huang, Yongping Gan, Yang Xia, Chu Liang, Wenkui Zhang and Jun Zhang *

College of Materials Science and Engineering, Zhejiang University of Technology, 310014 Hangzhou, China
* Correspondence: zhangjun@zjut.edu.cn

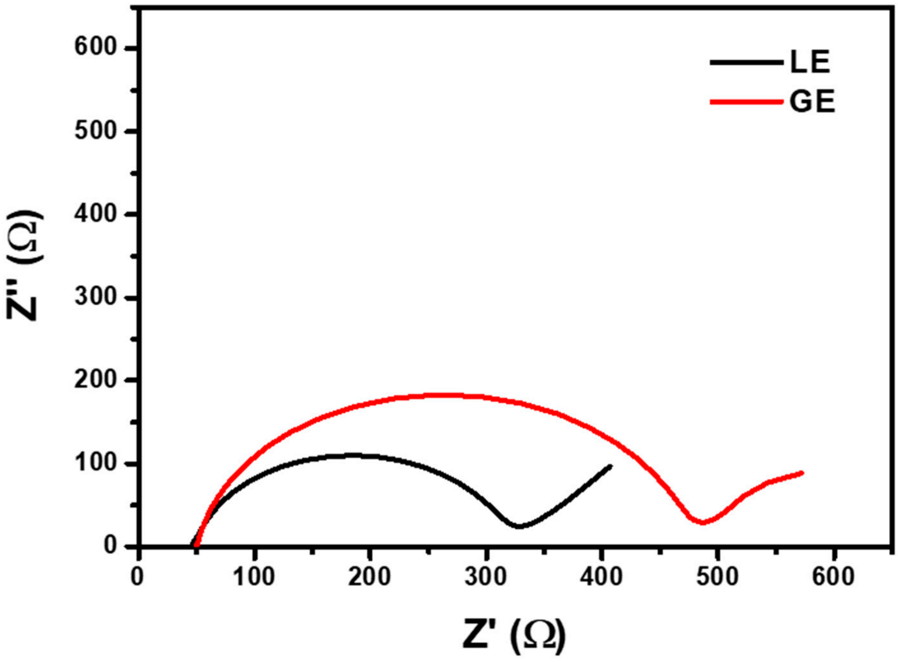

Figure S1. EIS spectra of the liquid electrolyte and gel electrolyte.

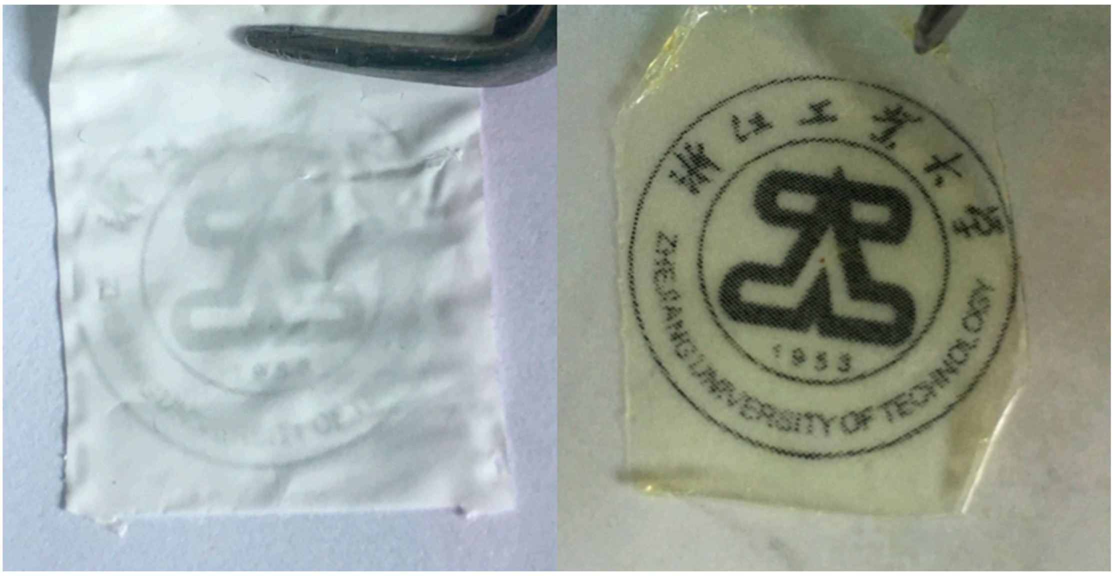

Figure S2. The photograph of PVDF-HFP membrane before and after soaking electrolyte.

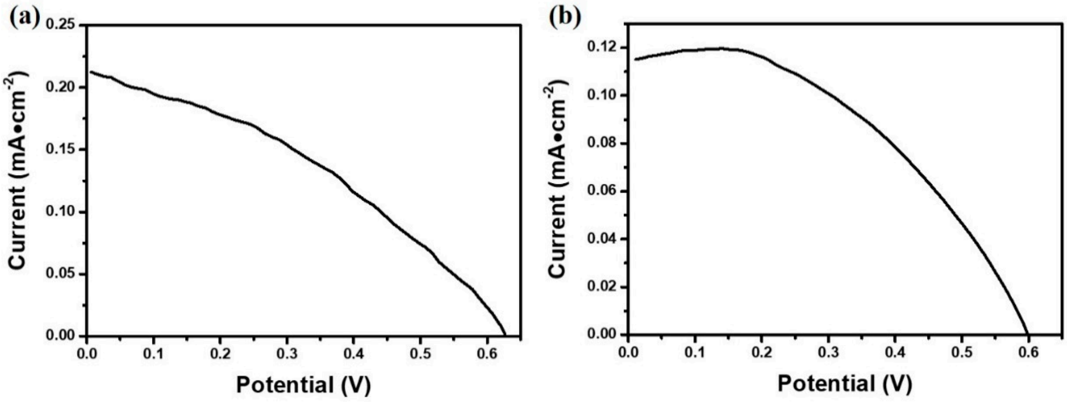

Figure S3. J-V curves of DSSC (**a**) and PECD (**b**) under the irradiation of 1000 W/m^2.

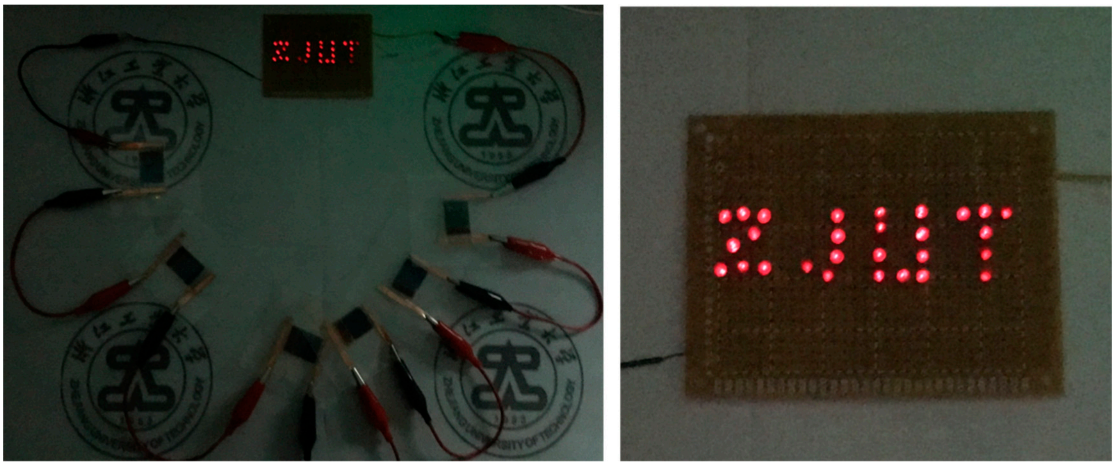

Figure S4. "ZJUT" composed by several red LEDs lit up by connected PECDs.

Table S1. Transmittance properties of PECD at 529 nm and 860 nm.

PECD state	T_{529nm}	ΔT	Tb/Tc	T_{860nm}	ΔT	Tb/Tc
1st colored	23%	40%	2.7	6%	65%	11.8
1st bleached	63%			71%		
1000th colored	44%	27%	1.6	19%	49%	3.6
1000th bleached	71%			68%		

© 2020 by the authors. Submitted for possible open access publication under the terms and conditions of the Creative Commons Attribution (CC BY) license (http://creativecommons.org/licenses/by/4.0/).
